# Motion Assessment for Accelerometric and Heart Rate Cycling Data Analysis

**DOI:** 10.3390/s20051523

**Published:** 2020-03-10

**Authors:** Hana Charvátová, Aleš Procházka, Oldřich Vyšata

**Affiliations:** 1 Faculty of Applied Informatics, Tomas Bata University in Zlín, 760 01 Zlín, Czech Republic; 2 Department of Computing and Control Engineering, University of Chemistry and Technology in Prague, 166 28 Prague 6, Czech Republic; A.Prochazka@ieee.org; 3 Czech Institute of Informatics, Robotics and Cybernetics, Czech Technical University in Prague, 160 00 Prague 6, Czech Republic; 4 Department of Neurology, Faculty of Medicine in Hradec Králové, Charles University, 500 05 Hradec Králové, Czech Republic; Oldrich.Vysata@fnhk.cz

**Keywords:** multimodal signal analysis, computational intelligence, machine learning, motion monitoring, accelerometers, classification

## Abstract

Motion analysis is an important topic in the monitoring of physical activities and recognition of neurological disorders. The present paper is devoted to motion assessment using accelerometers inside mobile phones located at selected body positions and the records of changes in the heart rate during cycling, under different body loads. Acquired data include 1293 signal segments recorded by the mobile phone and the Garmin device for uphill and downhill cycling. The proposed method is based upon digital processing of the heart rate and the mean power in different frequency bands of accelerometric data. The classification of the resulting features was performed by the support vector machine, Bayesian methods, *k*-nearest neighbor method, and neural networks. The proposed criterion is then used to find the best positions for the sensors with the highest discrimination abilities. The results suggest the sensors be positioned on the spine for the classification of uphill and downhill cycling, yielding an accuracy of 96.5% and a cross-validation error of 0.04 evaluated by a two-layer neural network system for features based on the mean power in the frequency bands 〈3,8〉 and 〈8,15〉 Hz. This paper shows the possibility of increasing this accuracy to 98.3% by the use of more features and the influence of appropriate sensor positioning for motion monitoring and classification.

## 1. Introduction

Recognition of human activities based on acceleration data [1,2,3,4,5,6] and their analysis by signal processing methods, computational intelligence, and machine learning, forms the basis of many systems for rehabilitation monitoring and evaluation of physical activities. Extensive attention has been paid to the analysis of these signals and their multimodal processing with further biomedical data [7,8] for feature extraction, classification, and human–computer interactions. Methods of motion detection and its analysis by accelerometers and global positioning systems (GPS) are also used for studies of physical activities including cycling [9,10,11,12,13,14], as assessed in this paper.

Sensor systems used for motion monitoring include wireless motion sensors (accelerometers and gyrometers) [15,16], camera systems (thermal, depth and color cameras) [9,17], ultrasound systems [18], and satellite positioning systems [12,13,14]. Specific methods are used for respiratory data processing [19] as well. There are many studies devoted to the analysis of these signals, markerless systems [20], and associated three-dimensional modelling.

There are very wide applications of motion monitoring systems, including gait analysis [21,22,23,24,25,26], motion evaluation [27,28,29,30,31,32], stroke patients monitoring [18,33], recognition of physical activities [34,35,36,37,38], breathing [39], and detection of motion disorders during sleep [40]. The combination of accelerometer sensors at different body locations in possible combination with GPS and geographical information systems is useful in improving movement monitoring of humans [41], assessing road surface roughness [10], and activity recognition [30] as well.

**Figure 1:**
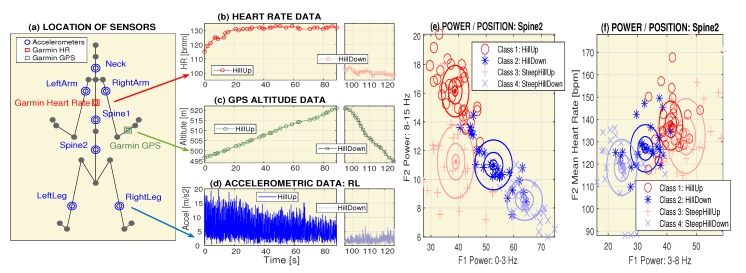
The principle of motion data acquisition and their processing, presenting (**a**) location of accelerometric and Garmin system sensors during cycling, (**b**–**d**) sample signals recorded by the heart rate sensor, Garmin position system, and the *RightLeg* accelerometric data while moving up and down, (**e**) distribution of the mean power in the frequency ranges 〈0,3〉 Hz and 〈8,15〉 Hz, and (**f**) distribution of the mean power in the frequency range 〈3,8〉 Hz and the mean heart rate for different cycling classes (route conditions) with cluster centers and ellipses showing multiples of the standard deviations.

The present paper is devoted to the use of these systems to recognize selected motion activities using data acquired by accelerometers in mobile phones [42,43,44] with positioning and heart rate (HR) data simultaneously recorded by the Garmin system [45,46]. The locations of the accelerometric and Garmin sensors used to monitor the motion and heart rate data are presented in Figure 1. The methods used for the data processing include data de-noising, statistical methods, neural networks [47], and deep learning [48,49,50,51] methods with convolutional neural networks.

The main goal of the present paper is the analysis of accelerometric and heart rate signals to contribute to monitoring physical activities and to the assesment of rehabilitation exercises [11,52]. Selected sensors were used for the analysis of data recorded during cycling in different conditions, extending the results recorded on the exercise bike [53,54]. The proposed mathematical tools include the use of neural networks [55], machine learning for pattern recognition, and the application of signal processing methods for data analysis to enable the monitoring of selected physiological functions.

## 2. Methods

### 2.1. Data Acquisition

Figure 1a presents the location of sensors for the acquisition of accelerometric, positioning, and heart rate data during cycling experiments with different loads. Both the mobile phone at different locations (for accelerometric data recording) and the Garmin system (for the simultaneous recording of GPS data and the heart rate) were used for data acquisition. Sample signals for uphill and downhill cycling are shown in Figure 1b–d.

The GPS and motion data (time stamps, longitude, latitude, altitude, cycling distance, and the cycling speed) were simultaneously measured by a Garmin fitness watch (Fenix 5S, Garmin Ltd., Schaffhausen, Switzerland). The heart rate data were acquired by a Garmin chest strap connected to a Garmin watch by the wireless data transmission technology. All data sets were acquired during the cycling experiments realised by a healthy and trained adult volunteer. Records were subsequently stored to the Garmin Connect website, exported in the specific Training Center (TCX) format (used for data exchange between fitness devices), converted to the comma-separated values (CSV), and imported into the MATLAB software for further processing.

A summary of the cycling segments for specific locations of the mobile phone used for accelerometric data acquisition is presented in Table 1.

The original mean sampling frequency was 142 Hz (changing in the range 〈15,300〉 Hz with the standard deviation STD = 114) for accelerometric data and 0.48 Hz (changing in the range 〈0.2,1〉 Hz, STD = 0.27) for heart rate data.

Table 2 presents the categories used for the classification. They were selected according to the profile of the terrain, its slope being evaluated by the Garmin GPS system. The individual categories include: (i) c(1)-*HillUp*; (ii) c(2)-*HillDown*; (iii) c(3)-*SteepHillUp*; and (iv) c(4)-*SteepHillDown* cycling.

A sample time segment of the modulus of the accelerometric data simultaneously recorded by the mobile phone at the selected location (the *RightLeg*) is presented in Figure 1d. All procedures involving human participants were in accordance with the ethical standards of the institutional research committee and with the 1964 Helsinki Declaration and its later amendments.

### 2.2. Signal Processing

The proposed data processing method included data analysis at first. The total number of 1293 cycling segments was reduced to 1254 segments in the initial step, to exclude those with the standard deviation of the speed higher than a selected fraction of its mean value. This process excluded 3% of the cycling segments with gross errors and problems on the cycling route, as specified in Table 1.

In the next step, the linear acceleration data without additional gravity components were processed. Their modulus $A_{q}\left(n\right)$ of the accelerometric data was evaluated from the components $Ax_{q}\left(n\right)$, $Ay_{q}\left(n\right)$, and $Az_{q}\left(n\right)$ recorded in three directions:

(1) $$A_{q}\left(n\right)=\sqrt{Ax_{q}{\left(n\right)}^{2}+Ay_{q}{\left(n\right)}^{2}+Az_{q}{\left(n\right)}^{2}}\;$$

for all values n=0,1,2,⋯,N-1 in each segment q=1,2,⋯,Q(pos)*N* values long, for all classes and at positions pos specified in Table 1. The Garmin data were used to evaluate the mean heart rate, cycling speed, and the mean slope in each segment. Owing to the slightly changing time period during each observation, the initial preprocessing step included the linear interpolation into a vector of uniformly spaced instants with the same endpoints and number of samples.

The processing of multimodal records ${\left\{s\left(n\right)\right\}}_{n=0}^{N-1}$ of the accelerometric and heart rate signals was performed by similar numerical methods. In the initial stage, their de-noising was performed by finite impulse response (FIR) filtering of a selected order *M*, resulting in a new sequence ${\left\{x\left(n\right)\right\}}_{n=0}^{N-1}$ using the relation

(2) $$x\left(n\right)=\sum_{k=0}^{M-1}b\left(k\right)\;s\left(n-k\right)\;$$

with coefficients ${\left\{b\left(k\right)\right\}}_{k=0}^{M-1}$ forming a filter of the selected type and cutoff frequencies. In the present study, the selected cutoff frequency $f_{c}=60$ Hz was used for the antialiasing low pass FIR filter of the order M=4. It allowed signal resampling for this new sampling frequency.

The accelerometric data were processed to evaluate the signal spectrum, covering the full frequency range of $\langle 0,f_{s}/2=30\rangle$ Hz related to the sampling theorem. The mean normalized power components in 4 sub-bands were then evaluated to define the features of each segment q=1,2,⋯,Q(pos) for each class and sensor position. The resulting feature vector F(:,q) includes in each of its columns *q* relative mean power values in the frequency bands $\langle f_{c1},f_{c2}\rangle$ Hz, which form a complete filter bank covering the frequency ranges of 〈0,3〉, 〈3,8〉, 〈8,15〉, and 〈15,30〉 Hz. The next row of the feature vector includes the mean heart rate in each segment q=1,2,⋯,Q(pos).

Each of the selected spectral features of a signal segment ${\left\{y\left(n\right)\right\}}_{n=0}^{N-1}$*N* samples long was evaluated using the discrete Fourier transform, in terms of the relative power PV in a specified frequency band $\langle f_{c1},f_{c2}\rangle$ Hz, as follows:

(3) $$PV\;=\;\frac{\sum_{k\in \Phi }{\left|Y(k)\right|}^{2}}{\sum_{k=0}^{N/2}{\left|Y(k)\right|}^{2}},\;Y\left(k\right)\;=\;\sum_{n=0}^{N-1}y\left(n\right)\;e^{-j\;kn\frac{2\pi }{N}}\;$$

where Φ is the set of indices for which the frequencies $f_{k}\;=\;\frac{k}{N}f_{s}\in \langle f_{c1},f_{c2}\rangle$ Hz.

Figure 1e,f presents selected features during different physical activities. Figure 1e shows the distribution of the mean power in the frequency ranges 〈0,3〉 Hz and 〈8,15〉 Hz, and Figure 1f presents the distribution of the mean power in the frequency range 〈3,8〉 Hz and the mean heart rate for different categories of cycling (route conditions) with cluster centers and ellipses showing multiples of the standard deviations.

The validity of a pair of features F1,F2 selected from the feature vector F(:,q) for all segments q=1,2,⋯,Q(pos) related to specific classes c(k) and c(l) and positions pos was evaluated by the proposed criterion $Z_{pos}\left(k,l\right)$ for cluster couples k,l defined by the relation:

(4) $$Z_{pos}\left(k,l\right)=\frac{D_{pos}\left(k,l\right)-ST_{pos}\left(k,l\right)}{Q_{pos}\left(k,l\right)}\;$$

where

(5) $$\begin{array}{ccc} D_{pos}\left(k,l\right) & = & dist(C_{pos}\left(k\right);C_{pos}\left(l\right)) \end{array}$$

(6) $$\begin{array}{ccc} ST_{pos}\left(k,l\right) & = & std\left(C_{pos}\left(k\right)\right)+std\left(C_{pos}\left(l\right)\right)\; \end{array}$$

using the Euclidean distance $D_{pos}\left(k,l\right)$ between the cluster centers $C_{k}$, $C_{l}$ of the features associated with classes *k* and *l*, respectively, and the sum $ST_{pos}\left(k,l\right)$ of their standard deviations. For well-separated and compact clusters, this criterion should take a value larger than zero.

**Figure 2:**
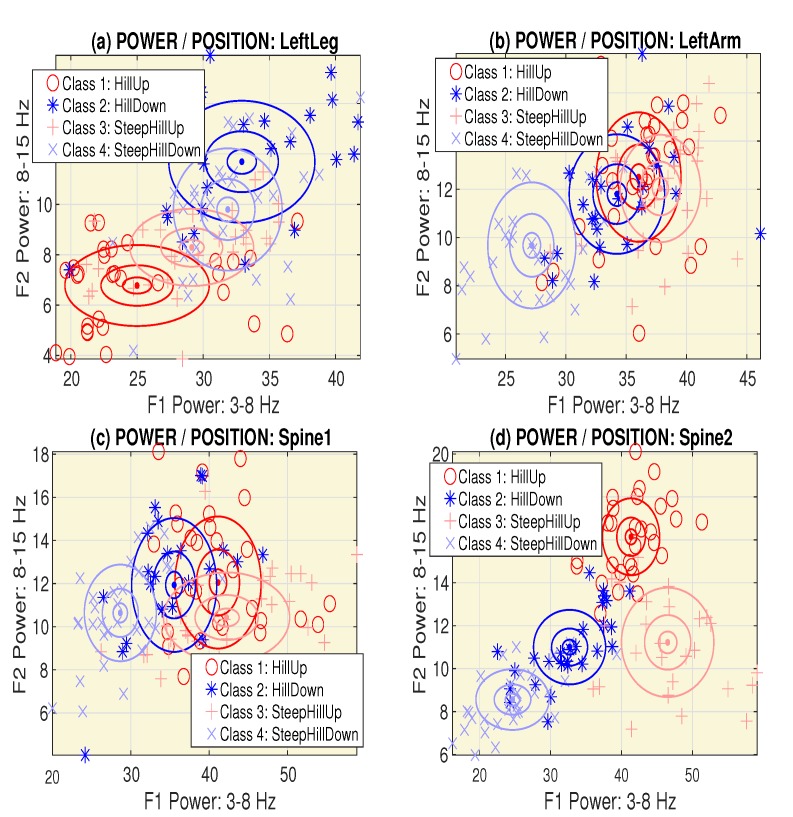
The distribution of the mean power in the frequency ranges 〈3,8〉 Hz and 〈8,15〉 Hz for the accelerometer at (**a**) the *LeftLeg*, (**b**) the *LeftArm*, (**c**) the upper *Spine1*, and (**d**) the lower *Spine2* position.

Signal analysis resulted in the evaluation of the feature matrix $P_{R,Q}$. The feature vector ${\left[p\left(1,q\right),p\left(2,q\right),\cdots ,p\left(R,q\right)\right]}^{\prime }$ in each of its columns includes both the mean power in specific frequency ranges and the mean heart rate. The target vector ${\mathrm{TV}}_{1,Q}={\left[t\left(1\right),t\left(2\right),\cdots ,t\left(Q\right)\right]}^{\prime }$ includes the associated terrain specification according to Table 2 with selected results in Figure 2. Different classification methods were then applied to evaluate these features.

### 2.3. Pattern Recognition

Pattern values in the feature matrix $P_{R,Q}$ and the associated target vector ${\mathrm{TV}}_{1,Q}$ were then used for classifying all *Q* feature vectors into separate categories. System modelling was performed by a support vector machine (SVM), a Bayesian method, the *k*-nearest neighbour method, and a neural network [22,55,56,57]. network Both the accuracies and the cross-validation errors were then compared with the best results obtained by the two-layer neural network.

The machine learning [57,58] was based on the optimization of the classification system with *R* = 5 input values (that corresponded with the features evaluated as the mean power in four frequency bands and the mean heart rate) and S2 output units in the learning stage. The target vector ${\mathrm{TV}}_{1,Q}$ was transformed to the target matrix $T_{S2,Q}$ with units in the corresponding class rows in the range 〈1,S2〉 to enable evaluating the probability of each class.

**Figure 3:**
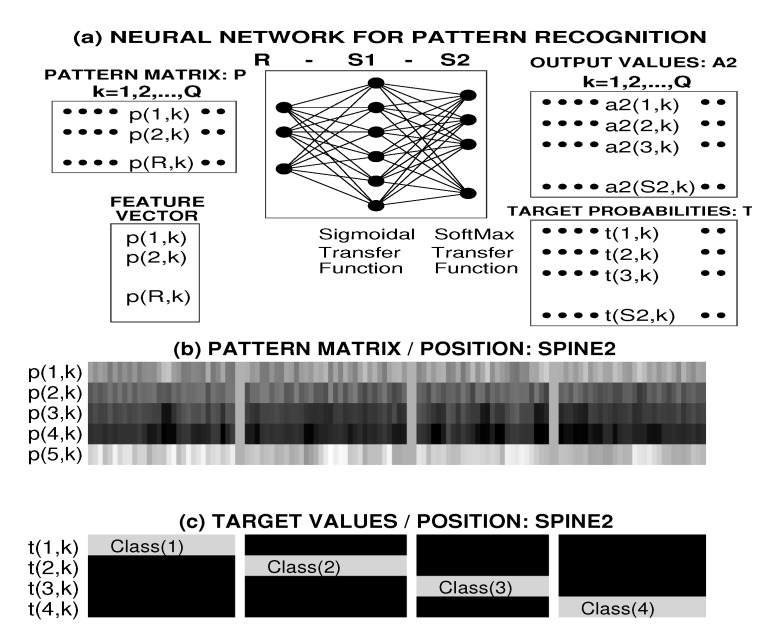
Pattern matrix classification using (**a**) the two-layer neural network with sigmoidal and softmax transfer functions, (**b**) feature matrix values for classification of segment features and a given sensor position (*Spine2*) into a selected number of classes, and (**c**) associated target matrix.

In the case of the neural network classification model, the pattern matrix $P_{R,Q}$ formed the input of the two-layer neural network structure with sigmoidal and softmax transfer functions presented in Figure 3a and used to evaluate the values by the following relations:

(7) $$\begin{array}{ccc} {A1}_{S1,Q}\;\; & = & \;\;f1({W1}_{S1,R}\;P_{R,Q},{b1}_{S1,1}) \end{array}$$

(8) $$\begin{array}{ccc} {A2}_{S2,Q}\;\; & = & \;\;f2({W2}_{S2,S1}\;{A1}_{S1,Q},{b2}_{S2,1}). \end{array}$$

For each column vector in the pattern matrix, the corresponding target vector has one unit element in the row pointing to the correct target value.

The network coefficients include the elements of the matrices ${W1}_{S1,R}$ and ${W2}_{S2,S1}$ and associated vectors ${b1}_{S1,1}$ and ${b2}_{S2,1}$. The proposed model uses the sigmoidal transfer function f1 in the first layer and the probabilistic softmax transfer function f2 in the second layer. The values of the output layer, based on the Bayes theorem [22], using the function

(9) $$\;f2\left(.\right)=\frac{exp(.)}{sum(exp(.))}$$

provide the probabilities of each class.

Figure 3b illustrates the pattern matrix formed by *Q* column vectors of *R* = 5 values representing the mean power in 4 frequency bands and the mean heart rate. Figure 3c presents the associated target matrix for a selected position of the accelerometric sensor.

Each column vector of grey shade pattern values was associated with one of the S2 different target values during the learning process.

The receiver operating characteristic (ROC) curves were used as an efficient tool for the evaluation of classification results. The selected classifier finds in the negative/positive set the number of true-negative (TN), false-positive (FP), true-positive (TP), and false-negative (FP) experiments.

The associated performance metrics [59] can then be used to evaluate:

- Sensitivity (the true positive rate, the recall) and specificity (the true negative rate):
  (10) $$SE=\frac{TP}{TP+FN},\;SP=\frac{TN}{TN+FP}\;;$$
- Accuracy:
  (11) $$ACC=\frac{TP+TN}{TP+TN+FP+FN}\;;$$
- Precision (the positive predictive value) and F1-score (the harmonic mean of the precision and sensitivity):
  (12) $$PPV=\frac{TP}{TP+FP},\;F1s=2\;\frac{PPV\cdot SE}{PPV+SE}\;.$$

Cross-validation errors [60] were then evaluated as a measure of the generalization abilities of classification models using the leave-one-out method.

## 3. Results

Table 3 presents a summary of the mean features for classification into 4 classes (c(1)-*SlopeUp*, c(2)-*SlopeDown*, c(3)-*SteepSlopeUp*, and c(4)-*SteepSlopeDown*) for different positions of the sensors and selected features (the relative mean power [%] in frequency ranges 〈0,3〉 Hz, 〈3,8〉 Hz, 〈8,15〉 Hz, 〈15,30〉 Hz, and the heart rate HR [bpm]).

**Figure 4:**
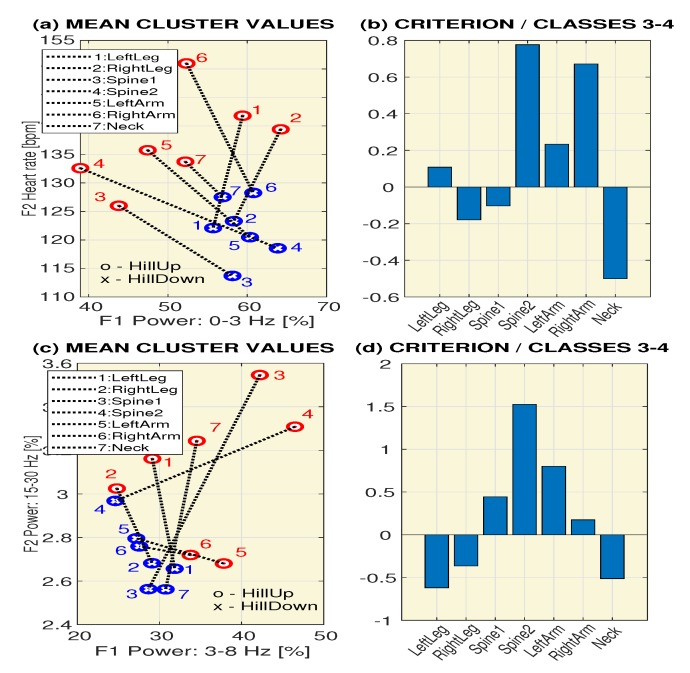
The evaluation of two classes (c(3)-*SteepHillUp* and c(4)-*SteepHillDown*) presenting cluster centers for different locations of sensors and associated values of the criterion function for features evaluated as (**a**,**b**) the mean power in the frequency range 〈0,3〉 Hz and the mean heart rate and (**c**,**d**) the mean power in frequency ranges 〈3,8〉 Hz and 〈15,30〉 Hz.

The validity of pairs of features F(i) and F(j) for separating classes $c_{k}$ and $c_{j}$ was then evaluated using the proposed criterion specified by Equation (4). Figure 4 presents the evaluation of two classes (c(3)-*SteepHillUp* and c(4)-*SteepHillDown*) with cluster centers for different locations of the sensors, and associated values of the criterion function for features evaluated as the mean power in the frequency range 〈0,3〉 Hz and the mean heart rate (Figure 4a,b) and the mean power in frequency ranges 〈3,8〉 Hz and 〈15,30〉 Hz (Figure 4c,d). The results presented here show that the highest discrimination abilities are possessed by a sensor located at the *Spine2* position.

**Figure 5:**
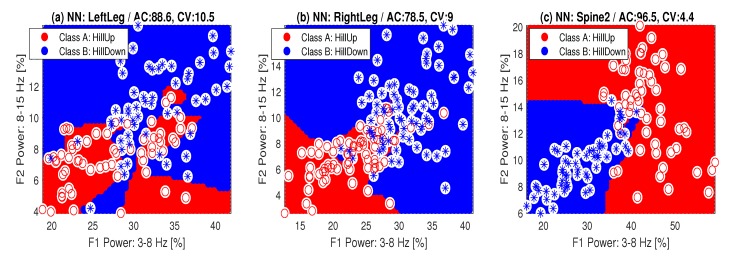
Classification of cycling segments into two categories (*A*-*HillUp* and *B*-*HillDown*) for two features evaluated as the mean power in the frequency ranges 〈3,8〉 Hz and 〈8,15〉 Hz for sensor locations in (**a**) the *LeftLeg*, (**b**) the *RightLeg*, and (**c**) *Spine2* with accuracy (AC [%]) and cross-validation (CV) errors.

Figure 5 presents the classification of cycling segments into two categories (*A*-*HillUp* and *B*-*HillDown*) for two features evaluated as the mean power in the frequency ranges 〈3,8〉 Hz and 〈8,15〉 Hz for the sensor locations (a) the *LeftLeg*, (b) *RightLeg*, and (c) *Spine2* with accuracy (AC) and cross-validation (CV) errors. More detailed results of this classification are presented in Table 4. Its separate rows present the accuracy AC [%] and cross-validation errors for the classification of class *A* (*HillUp*: c(1)+c(3)) and class *B* (*HillDown*: c(2)+c(4)) for different locations of the sensors, chosen features (F1—frequency range 〈3,8〉 Hz, F2—frequency range 〈8,15〉 Hz) and selected classification methods. The highest accuracy and the lowest cross-validation errors were achieved by the *Spine2* location of the accelerometric sensors and all classification methods.

Table 5 presents the accuracy AC [%], specificity (TNR), sensitivity (TPR), F1-score (F1s), and cross-validation errors CV for classification into classes *A* and *B* by the neural network model for different locations of sensors and 5 features F1–F5 including the power in all four frequency bands and the mean heart rate in each cycling segment. The highest accuracy, 98.3%, was achieved again for the *Spine2* position of the accelerometric sensor with the highest F1-score of 98.2% as well.

**Figure 6:**
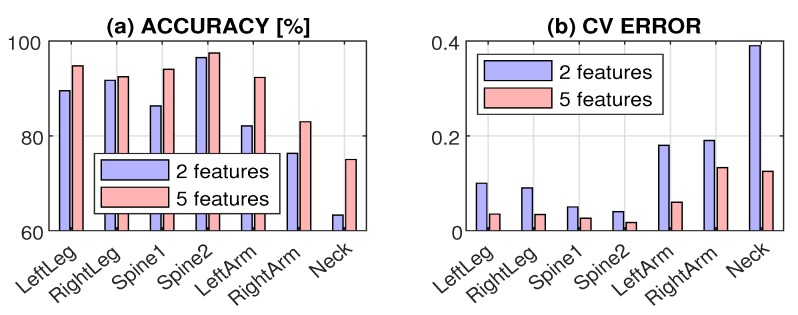
Comparison of neural network classification presenting (**a**) accuracy and (**b**) cross-validation (CV) error using two and five features for different positions of the accelerometer.

The comparison of neural network classification for two and five features is presented in Figure 6 related to Table 4 and Table 5. Cross-validation errors are evaluated by the leave-one-out method in all cases. Figure 6a shows that there is an increase in the accuracy by 6.17% on average that is most significant for locations with the lowest accuracy, including the *arm* and *neck* positions. In a similar way, an increase in the number of features from two to five decreased the cross-validation error on average by 8.72%, as presented in Figure 6b. This decrease was most significant for locations with the lowest accuracy and the highest error, which included the *arm* and *neck* positions again.

## 4. Conclusions

This paper has presented the use of selected methods of machine learning and digital signal processing in the evaluation of motion and physical activities using wireless sensors for acquiring accelerometric and heart rate data. A mobile phone was used to record the accelerometric data at different body positions during cycling, under selected environmental conditions.

The results suggest that accelerometric data and the associated signal power in selected frequency bands can be used as features for the classification of different motion patterns to recognize cycling terrain and downhill and uphill cycling.

The proposed criterion selected the most appropriate position for classification of motion: it was the *Spine2* position. All classification methods, including a support vector machine, a Bayesian method, the *k*-nearest neighbour method, and a two-layer neural network, were able to distinguish specific classes with an accuracy higher than 90%. The best results were achieved by the two-layer neural network and *Spine2* position with an accuracy of 96.5% for two features, which was increased to 98.3% for five features.

These results correspond with those achieved during cycling on a home exercise bike [4,54] with different loads and additional sensors, including thermal cameras as well.

It is expected that further studies will be devoted to the analysis of more extensive data sets acquired by new non-invasive sensors, enabling the detection of further motion features with higher sampling frequencies. Special attention will be devoted to further multichannel data processing tools and deep learning methods with convolutional neural networks to improve the possibilities of remote monitoring of physiological functions.

## Figures and Tables

**Table 1:** Summary of cycling segments of individual positions P(pos) used for classification.

Position Index	Position Name	Number of Segments	
*pos*	*P(pos)*	Used: *Q(pos)*	Rejected
1	*LeftLeg*	180	6
2	*RightLeg*	210	9
3	*Spine1*	177	9
4	*Spine2*	174	6
5	*LeftArm*	177	3
6	*RightArm*	198	0
7	*Neck*	177	6
TOTAL NUMBER:		1293	39

**Table 2:** The mean slope *S* [%] of the terrain segments, and its standard deviation (STD), as recorded by the Garmin GPS system.

Class	*S[%]*	STD
c(1)-*HillUp*	10.3	3.3
c(2)-*HillDown*	−9.4	3.9
c(3)-*SteepHillUp*	19.8	3.4
c(4)-*SteepHillDown*	−18.7	3.4

**Table 3:** Mean features for classification into 4 classes (c(1)-*SlopeUp*, c(2)-*SlopeDown*, c(3)-*SteepSlopeUp*, and c(4)-*SteepSlopeDown*) for different positions of sensors and selected features (the percentage mean power $F_{\langle 0,3\rangle }$, $F_{\langle 3,8\rangle }$, $F_{\langle 8,15\rangle }$, and $F_{\langle 15,30\rangle }$, and the heart rate HR [bpm]).

Position	Feature	*c(1)*		*c(2)*		*c(3)*		*c(4)*	
		Mean	STD	Mean	STD	Mean	STD	Mean	STD
*LeftLeg *	$F_{\langle 0,3\rangle }$ [%]	66	7	52	7	59	6	56	7
	$F_{\langle 3,8\rangle }$ [%]	25	5	33	6	29	5	32	4
	$F_{\langle 8,15\rangle }$ [%]	7	2	12	2	8	2	10	2
	$F_{\langle 15,30\rangle }$ [%]	2	1	3	1	3	1	3	2
	HR[bpm]	137	22	129	19	142	26	122	17
*RightLeg *	$F_{\langle 0,3\rangle }$ [%]	71	5	51	7	64	6	58	6
	$F_{\langle 3,8\rangle }$ [%]	21	4	34	4	25	4	29	4
	$F_{\langle 8,15\rangle }$ [%]	6	1	12	3	8	2	10	2
	$F_{\langle 15,30\rangle }$ [%]	2	1	3	2	3	1	3	1
	HR[bpm]	142	20	125	23	139	26	123	24
*Spine1 *	$F_{\langle 0,3\rangle }$ [%]	44	7	50	8	44	10	58	7
	$F_{\langle 3,8\rangle }$ [%]	41	6	36	5	42	8	29	5
	$F_{\langle 8,15\rangle }$ [%]	12	3	12	3	10	2	11	2
	$F_{\langle 15,30\rangle }$ [%]	3	2	3	2	4	1	3	2
	HR[bpm]	146	16	130	18	126	25	114	18
*Spine2 *	$F_{\langle 0,3\rangle }$ [%]	39	5	53	7	39	7	64	7
	$F_{\langle 3,8\rangle }$ [%]	41	4	33	5	47	7	25	5
	$F_{\langle 8,15\rangle }$ [%]	16	2	11	2	11	3	9	1
	$F_{\langle 15,30\rangle }$ [%]	4	1	4	1	3	1	3	0
	HR[bpm]	137	11	127	11	133	17	119	13
*LeftArm *	$F_{\langle 0,3\rangle }$ [%]	48	6	50	6	47	5	60	6
	$F_{\langle 3,8\rangle }$ [%]	36	4	34	4	38	3	27	4
	$F_{\langle 8,15\rangle }$ [%]	13	3	12	2	12	2	10	3
	$F_{\langle 15,30\rangle }$ [%]	3	2	4	2	3	2	3	1
	HR[bpm]	144	13	140	12	136	18	120	16
*RightArm *	$F_{\langle 0,3\rangle }$ [%]	47	6	50	7	52	5	61	5
	$F_{\langle 3,8\rangle }$ [%]	38	4	36	5	34	3	28	3
	$F_{\langle 8,15\rangle }$ [%]	12	2	11	2	11	2	9	2
	$F_{\langle 15,30\rangle }$ [%]	3	1	3	2	3	2	3	1
	HR[bpm]	153	11	138	11	151	20	128	14
*Neck *	$F_{\langle 0,3\rangle }$ [%]	49	7	53	8	52	7	57	5
	$F_{\langle 3,8\rangle }$ [%]	36	4	34	5	35	6	31	4
	$F_{\langle 8,15\rangle }$ [%]	12	3	11	4	10	2	10	2
	$F_{\langle 15,30\rangle }$ [%]	4	1	3	2	3	1	3	1
	HR[bpm]	145	13	142	12	134	18	128	14

**Table 4:** Accuracy (AC [%]) and cross-validation errors for classification of class *A* (*HillUp*: c(1)+c(3)) and class *B* (*HillDown*: c(2)+c(4)) for different locations of sensors, chosen features (F1—frequency range 〈3,8〉 Hz, F2—frequency range 〈8,15〉 Hz) and selected classification methods: support vector machine (SVM), Bayes, 5-nearest neighbour (5NN) and neural network (NN) methods (with the highest accuracy and the lowest cross-validations errors in bold).

Position	Accuracy AC [%]				Cross-validation Error			
	SVM	Bayes	5NN	NN	SVM	Bayes	5NN	NN
*LeftLeg*	81.6	75.4	85.1	89.5	0.22	0.25	0.20	0.10
*RightLeg*	84.7	82.6	86.8	91.7	0.15	0.17	0.19	0.09
*Spine1*	86.3	77.8	84.6	86.3	0.18	0.23	0.21	0.05
*Spine2*	**92.1**	**89.5**	**93.7**	**96.5**	**0.09**	**0.11**	**0.07**	**0.04**
*LeftArm*	82.1	78.6	82.9	82.1	0.24	0.21	0.23	0.18
*RightArm*	75.6	65.9	79,3	76.3	0.37	0.36	0.39	0.19
*Neck*	61.7	63.3	70.8	63.3	0.63	0.37	0.49	0.39

**Table 5:** Accuracy (AC [%]), specificity (TNR [%]), sensitivity (TPR [%]), F1-score (F1s [%]), and cross-validation (CV) errors for classification into classes *A* and *B* by the neural network model for different location of sensors and features F1–F5 (with the highest accuracy and the lowest cross-validations errors in bold).

Position	*AC [%]*	*TNR* *[%]*	*TPR [%]*	*F1s [%]*	*CV*
*LeftLeg*	93.3	92.1	94.7	93.1	0.083
*RightLeg*	95.0	97.3	91.2	94.5	0.042
*Spine1*	96.7	96.8	96.5	96.5	**0.017**
*Spine2*	**98.3**	**98.4**	**98.2**	**98.2**	0.042
*LeftArm*	93.3	95.2	91.2	92.9	0.067
*RightArm*	94.8	93.9	95.7	94.9	0.030
*Neck*	95.8	95.2	96.6	95.7	0.033
